# Perinatal Outcome in Overweight Women: An Audit

**DOI:** 10.7759/cureus.48033

**Published:** 2023-10-31

**Authors:** Neeru Malik, Neeraj Sharma, GP Kaushal, Dakshika Lochan, Sandhya Jain, Manju Ghotiya, Nikita Madaan

**Affiliations:** 1 Department of Obstetrics and Gynaecology, Dr. Baba Saheb Ambedkar Medical College & Hospital, Delhi, IND; 2 Department of Pediatrics, Dr. Baba Saheb Ambedkar Medical College & Hospital, Delhi, IND; 3 Department of Obstetrics and Gynaecology, Employees' State Insurance Corporation-Post Graduate Institute of Medical Science & Research (ESIC-PGIMSR), Delhi, IND

**Keywords:** induction of labor outcome, obstetric labor complications, pregnancy outcome, body mass index: bmi, maternal obesity

## Abstract

Background: Obesity in pregnancy is associated with a myriad of well-documented complications. However, the outcomes of pregnancy in overweight females, who are not classified as obese, have not been studied. The aim of the study was to assess foeto-maternal outcomes in primigravida who are overweight and compare them to normal-weight patients.

Material and methods: This was a prospective observational cohort study and included primigravida with full-term gestation (between 38 and 42 weeks), with a single live foetus in vertex presentation, who were admitted for labour induction. Based on pre-pregnancy weight, patients were divided into normal weight (body mass index, BMI<23kg/m^2^) and overweight (BMI≥23kg/m^2 ^and<25kg/m^2^) categories labelled as groups A and B, respectively. Data was collected for gestational age, demographics (age, education, occupation), and obstetric and labour-related parameters per pre-designed proforma. Parameters included were the reason for induction, number of doses of prostaglandin E2 (PGE2) gel used, duration of labour, induction to delivery interval, and mode of birth- operative/ non-operative. Data was also collected for peri-partum maternal complications, neonatal Apgar score, and need for Neonatal Intensive Care Unit (NICU) admissions.

Results: One hundred and fifty patients were recruited in the study and divided based on weight into two groups- 115 in Group A (normal weight) and 35 in Group B (overweight). Compared to Group A, a higher proportion of patients in Group B needed a third dose of PGE2 gel (n=24, 20.8% vs n=18, 51.4%). Also, more patients in Group B had an induction to delivery time of longer than 30 hours (n=7, 20% vs n=5, 4.3%) and had a higher incidence of failed induction needing caesarean section (n=9, 25.7% vs n=13, 11.3%). Neonates born to overweight mothers had a poor Apgar score at 1 min. However, on reassessment, Apgar improved at 5 minutes, and no statistically significant difference was seen for admission to NICU- 5.7% (n=2) in Group B vs 1.7% (n=2) in Group A

Conclusion: Pregnancy in overweight females is associated with prolonged labour, higher instances of failed induction, and poor neonatal outcomes at initial assessment. Thus, perinatal counselling and management should focus on weight control while also planning appropriate strategies for monitoring and treating pregnancy-related complications if weight control measures fail. Although obesity is the main focus of research, we suggest including overweight but non-obese females in such studies as they have similar adverse outcomes and complications.

## Introduction

Obesity, defined as a body mass index (BMI) of more than 23kg/m^2^ for the south-east population [1], is a significant disease worldwide, with its prevalence tripling in the last four decades [2]. The percentage of overweight or obese women has climbed from 20.6% in 2016 to 24% in 2021, according to a survey in India [3]. This increase represents a challenging problem that multidimensionally affects the patient's health, especially women's reproductive health.

Obesity affects various aspects of women's health, such as fertility, lifetime hormonal changes, polycystic ovary syndrome, risk of heart disease, diabetes, and breast cancer [4]. It is frequently associated with pregnancy and can lead to multiple foeto-maternal complications across all stages of pregnancy, ranging from gestational diabetes mellitus (GDM), pregnancy-induced hypertension, pre-eclampsia (PE) to complicated labour, and post-partum complications like the risk of venous thromboembolism [5]. Further, adverse outcomes seen in foetus include macrosomia, stillbirth, prematurity and intrauterine foetal death. The effect of obesity on the foetus may extend beyond pregnancy into neonatal and childhood periods [6].

A meta-analysis of cohorts from Europe, North America, and Australia revealed that obesity raised the risk of GDM by three times and the risk of PE by two times. Obesity is a common risk factor for both GDM and PE. PE is independently correlated with both obesity and GDM, and the two have a more substantial effect when combined. A population-based retrospective cohort study from Sweden examined the effect of pre-pregnancy BMI on the development of GDM. The study included 13,057 GDM patients who required treatment and found that obesity increased the risk of developing GDM [7].

Obesity in the mother can directly affect the mode of birth and post-natal morbidity. Obese women are known to require labour induction twice as frequently as non-obese pregnant women. The probability of delay in the first stage of labour is 1.5 to 3 times higher in obese women. In addition, obese women have a two to three-fold higher risk of undergoing a caesarean section, with the most frequent cause being uterine inertia [8].

On reviewing the literature, very few studies have been done in India to see the correlation between the outcomes of labour and maternal BMI. Our study was done to compare obstetrical outcomes beyond 38 weeks of gestation in overweight versus normal weight primigravida, precisely- the number of prostaglandin E2 (PGE2) gel doses required for induction (0.5mg intracervical given 6 hourly for a maximum of three doses), induction to delivery time, frequency of caesarean section, and associated foeto-maternal complications.

## Materials and methods

Study design and settings

A one-year prospective observational cohort research was conducted at a tertiary care facility (Dr. Baba Saheb Ambedkar Medical College & Hospital) in Delhi.

Inclusion and exclusion criteria

After receiving informed consent, booked primigravida with full-term gestation (between 38 and 42 weeks), with a single live foetus, and vertex presentation who were admitted for labour induction were included in the study. Patients who had previously undergone uterine surgery, had ante-partum haemorrhage, skeletal deformities, multiple pregnancies, and foetal distress, were unsure about their last menstrual period (LMP), and had not undergone an early pregnancy scan (6-9 weeks) were excluded from the study. The study also excluded the women with irregular menstrual cycles and women whose pre-pregnancy or early pregnancy weight was unknown.

Study protocol

Informed consent was obtained from each patient in their own language after the study was approved by the Dr. Baba Saheb Ambedkar Medical College Institutional Ethics and Research Board with approval number F5(59)2017/BSAH/DNB/13421. A thorough history was collected, including socio-demographic information, parity, the duration of pregnancy, and general physical and obstetric examinations. The patient’s weight was taken as pre-pregnancy weight in kilograms and height in metres [9]. BMI was calculated by weight (Kg)/ height (metres)^2^. Menstrual history was used to estimate the gestational age. Gestational age was determined from a first-trimester ultrasound performed between 6 and 9 weeks after conception. The first-trimester ultrasound scan was done by a consultant radiologist, specially trained in obstetric scanning.

Based on their pre-pregnancy weight, patients were divided into groups: Group A with BMI<23 Kg/m^2 ^and Group B with BMI≥23kg/m^2^ and<25kg/m^2^. Group A had 115 patients, while Group B had 35 patients, totalling 150 patients in the study. General examination was done by the standardised method in all patients along with obstetric examination, vaginal examination, pelvic assessment, and bishop score. PGE2 gel was used for induction in all patients, 0.5mg given intracervically six hourly up to a maximum of three doses. When a patient entered the active stage of labour, oxytocin was utilised as an augmentation strategy if needed. The number of doses of PGE2 gel required, mode of delivery, induction to delivery interval, and foetal Apgar score at 1 and 5 minutes were all used to measure outcomes.

Sample size calculation

According to a Center for Disease Control (CDC) report published in 2020, the caesarean section rate in normal-weight females is around 20%-25%, while in overweight females it is around 45%. It goes up to 52.3% in class 3 obese females [10]. The ratio of subjects to be recruited in two groups (normal: overweight) was kept at 3:1 due to less prevalence of overweight primigravida. Taking the above values along with alpha of 0.05 and power of 80%, the calculated sample size was 136 females divided in 3:1 ratio. Considering that 10% of subjects might withdraw consent or drop out due to other reasons, the final sample size came out to be 150 subjects.

**Figure 1:**
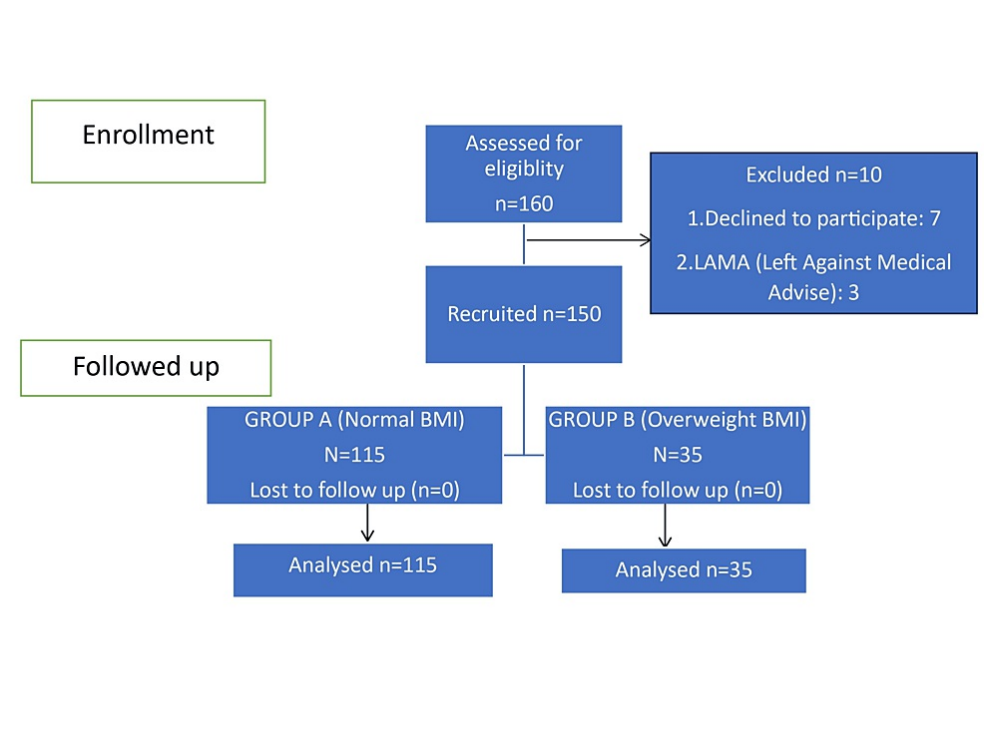
STROBE diagram of the study population and phases STROBE: STrengthening the Reporting of OBservational studies in Epidemiology

Staying true to the above calculations, we enrolled 150 females in our study with Group A (normal BMI), including 115 females and Group B (overweight), including 35 females (Figure 1).

Statistical analysis

 IBM SPSS Statistics for Windows, Version 21 (Released 2012; IBM Corp., Armonk, New York, United States) was used to analyze once the data had been imported into a Microsoft Excel (Microsoft Corporation, Redmond, Washington) spreadsheet. Continuous variables were reported as mean SD and median, whereas categorical variables were presented as number and percentage (%). Given that the data sets were not normally distributed, quantitative variables were compared between the two groups using the Mann-Whitney test. The Chi-Square and Fisher's exact tests were used to determine the correlation of qualitative variables. Statistical significance was defined as a p-value<0.05.

## Results

The study included 150 women who met inclusion criteria, of which 115 (76.66%) were within normal BMI (Group- A), and 35 (23.33%) women (Group- B) were in the overweight category (Table 1).

**Table 1:** Distribution and association of study participants according to BMI Data represented as numbers and (%)

BMI	Number of participants
GROUP A (BMI<23 kg/m^2^)	115 (76.66%)
GROUP B (23kg/m^2^ ≤BMI <25kg/m^2^)	35 (23.33%)

Table 2 shows the socio-demographic distribution of the study population. The socio-demographic parameters included patient age, level of education, occupation, and gestational age. Although there were slight differences between the groups, none achieved statistical significance; overall, both groups were comparable.

**Table 2:** Comparison of groups based on socio-demographic data Data represented as numbers and (%) p-value <0.05 is significant

Socio-demographic parameters		Normal weight (n=115)	Overweight (n=35)	Total	p-value
Age (years)	≤25 years	75 (65.22%)	18 (51.42%)	93 (62%)	0.143
	>25 years	40 (34.78)	17 (58.57 %)	57 (38%)	
Education	Uneducated	60 (52.17%)	11 (31.42%)	71 (47.33%)	0.081
	9^th ^-12^th ^standard	38 (33.04%)	15 (42.85%)	53 (35.33%)	
	Graduate	17 (14.78%)	9(25.71%)	26 (17.33%)	
Occupation	Housewife	86 (74.78%)	26 (74.28%)	112(74.66%)	0.942
	Laborer	5 (4.34%)	2 (5.71%)	7 (4.66%)	
	Professional	24 (20.86%)	7 (20%)	31 (20.66%)	
Gestational age (weeks + days)	38-38 +6	29 (25.21%)	11 (31.42%)	40 (26.66%)	0.426
	39-39+6	33 (28.69%)	13 (37.14%)	46 (30.66%)	
	40-40+6	31 (26.95%)	8 (22.85%)	39 (26%)	
	41-41+6	20 (17.39%)	2 (5.71%)	22 (14.66%)	
	42-42+6	2 (1.73%)	1 (2.85%)	3 (2%)	

In labour-related parameters, we recorded indications for labour induction, number of PGE2 gel doses used, duration of labour, mode of birth, peripartum complications, neonatal Apgar score at 1 and 5 minutes, and Neonatal Intensive Care Unit (NICU) admissions. The indications for labour induction included GDM, intra-hepatic cholestasis of pregnancy, foetal growth restriction, oligohydramnios, post-dated pregnancy, pre-eclampsia, premature rupture of membranes. The indications for labour induction and their relative frequencies were similar between groups.

As for the number of PGE2 gel doses used, most women delivered in less than three applications of PGE2 gel. Third gel application was required in 20.8% (N=24) of women in group A and 51.4% (N=18) in group B. Significantly greater dosages of PGE2 gel were needed in Group B compared to Group A (p-value <0.001).

In Group A, 26 women (22.6%) delivered within 10 hours of induction, while only two women (5.7%) in Group B delivered simultaneously. The difference was statistically significant, with a p-value of 0.024. Although 138 women (92%) in the study delivered within 30 hours of induction, 12 patients prolonged labour to more than 30 hours. These 12 patients included 5 (4.34%) from group A and 7 (20%) from group B (p-value 0.002). These findings show that labour is significantly prolonged in overweight women.

Regarding the mode of birth, the rate of caesarean section (CS) in Group A was 11.30% (13). In comparison, 25.71% (9) in Group B. Group B had significantly more patients undergoing CS (absolute difference- 14.4%) with a p-value of 0.034. Predominant indications for caesarean delivery were foetal distress and failed induction. 

The Apgar score at 1 minute was more than 7 for 96.52% (111) of neonates of Group A and 85.71% (30) of neonates of Group B mothers. This difference was statistically significant, with a p-value of 0.018. However, the Apgar score improved at 5 minutes and was not significantly different. No statistically significant intergroup difference was seen in the neonatal intensive care unit (NICU) admission rates. Four neonates were admitted to NICU; 2 belonged to mothers in Group A (1.73%), while 2 from mothers in Group B (5.72%), p-value- 0.20.

Table 3 summarises the above outcome data of both the groups.

**Table 3:** Comparison between groups regarding the delivery outcome, post-partum complications, and neonatal outcomes Data represented as frequency and (%) GDM: Gestational diabetes mellitus, IHCP: intra-hepatic cholestasis of pregnancy, IUGR: intra-uterine growth retardation, MSL: meconium-stained liquor, NICU: neonatal intensive care unit, PPH: post-partum haemorrhage, PROM: premature rupture of membranes, PGE2: prostaglandin E2 *p-value <0.05 is significant

			Normal weight (n=115)	Overweight (n=35)	Total	P- value
Number of PGE2 gel doses used (0.5 mg Intracervical gel 6 hourly)	1		36 (31.3%)	6 (17.14%)	37	0.26
	2		55(47.8%)	11 (31.43%)	71	0.33
	3		24 (20.8%)	18 (51.43%)	42	<0.001*
Indication	GDM		1 (0.86%)	2 (5.7%)	3	0.073
	IHCP		5 (4.34%)	1 (2.85%)	6	0.693
	IUGR		1 (0.86%)	1 (2.85%)	2	0.369
	Oligohydramnios		5(4.34%)	2 (5.7%)	7	0.737
	Post-datism		84 (73%)	26 (74.28%)	110	0.884
	Pre-Eclampsia		3 (2.6%)	1 (2.85%)	4	0.936
	PROM		16 (13.91%)	2 (5.7%)	18	0.191
Induction to delivery interval (hours)	≤ 10 hrs		26 (22.6%)	2(5.7%)	28	0.024*
	10hrs 1 min – 20 hrs		63 (54.78%)	13 (37.1%)	76	0.067
	20 hrs 1 min - 30 hrs		21 (18.26%)	14 (40%)	35	0.007*
	>30 hours		5 (4.34%)	7(20%)	12	0.002*
Mode of birth	Caesarean section (CS) Indication:	Total	13 (11.30%)	9 (25.71%)	22	0.034*
		Failed induction	5 (4.34%)	7 (20%)	12	0.068
		Foetal distress	8 (6.95%)	2 (5.71%)	10	
	Vaginal delivery		102 (88.69%)	26 (74.28%)	128	0.066
Complications (if any)	3^rd^-degree perineal tear		1 (0.86%)	1 (2.85%)	2	0.945
	Hyperstimulation		2 (1.73%)	1 (2.85%)	3	
	MSL		3 (2.6%)	1 (2.85%)	4	
	PPH		2 (1.73%)	1 (2.85%)	3	
No complication			107 (93.4%)	31 (88.50%)		0.393
Apgar Score At 1 minute	<7		4(3.47%)	5 (14.28%)	138	0.018*
	≥7		111(96.52%)	30 (85.71%)	141	
At 5 minutes	<7		3 (2.60%)	3(8.57%)	6	0.114
	≥7		112(97.39%)	32 (91.42%)	144	
NICU admission	Yes		2 (1.73%)	2 (5.72%)	4	0.201
	No		113 (98.26%)	33(94.28%)	146	

## Discussion

Obesity is a health problem reaching epidemic proportions. The percentage of overweight or obese women has climbed from 20.6% in 2016 to 24% in 2021, according to a survey in India [3]. Obesity during pregnancy is highly prevalent and increases obstetrical risks and the possibility of caesarean section. Thus, it is crucial to focus on its impacts on labour.

Our study shows that pregnancy in overweight women has an impact on both labour as well as the mode of birth. This is represented by a highly significant CS rate in the overweight (n=9, 25.71%) versus normal BMI group (n=13, 11.30%). The odds for birth by CS in the overweight group were 2.71 times that in the normal BMI group. These findings are supported by an earlier study by Khalifa et al., in which the CS rate in the normal BMI group was 13%, while higher CS rates were observed in the overweight and obesity class 1 groups at 18% and 40%, respectively [6]. In another retrospective cohort study done in 2022 by Bjorklund et al., according to the BMI group, there was a considerable rise in the likelihood of CS following the induction of labour in women with high BMI that is 18.4-24.1% of deliveries were by CS. This observed difference is likely because obesity is associated with a significant prolongation of labour, higher rates of non-progression, and slower cervical ripening, often interpreted as primary dystocia leading to unnecessary interventions. Also, a higher risk of foetal macrosomia and shoulder dystocia increases the incidence of emergency CS [11].

We found evidence in our study that labour induction appears to take more time as maternal BMI increases and requires an increased number of PGE2 gel doses for cervical ripening. It was observed that 22.6 % (26) of women in Group A delivered in less than 10 hours while only 5.7% (2) of women in Group B delivered simultaneously, the difference of which is statistically significant (p-value=0.024). Most women needed less than three doses of PGE2 gel to deliver. The third dose of PGE2 gel application was required in only 20.8% (n=24) of women in Group A, but in Group B, the number was significantly higher at 51.4% (n=18). Also, the duration of labour was significantly prolonged in the overweight group, with 20% (n=7) of patients in this group having a labour duration of more than 30 hours compared to 4.34 % (n=5) in the normal BMI group. Our results are supported by evidence from a study by Dammer et al., which investigated the impact of elevated BMI on the progression of labour. The study found a significant correlation between BMI and duration of labour, where the mean induction to the delivery interval in the normal weight group was 25.8 hours compared to 27.81 hours in obese group [12].

Pathophysiology for the above observations remains unclear, but research suggests that decreased oxytocin receptor sensitivity, endocrine factors and reduced uterine contractility may be the causes of prolonged labour in obese patients. Moreover, immature cervix, excessive fat build-up, and delayed natural labour are likely to raise the incidence of poor Bishop score in obese females, further increasing the chances of failed induction, prolonged labour and higher CS rates [13].

Prolongation of labour and increase in CS rates in overweight women is due to the interplay of multiple factors. A meta-analysis done in 2018 found that maternal obesity slows down the progression of labour. Also, it is related to longer gestational periods, the need for higher prostaglandin doses, fewer instances of successful cervical ripening procedures, a higher requirement of oxytocin, and a longer duration of labour following oxytocin administration. Increased incidence of CS was due to foetal macrosomia, failed induction, foetal distress, and the increased risk of shoulder dystocia [14]. The findings were only partially replicated in our study, likely due to two possible reasons. One, our study had a small sample size, which may have limited the entire spectrum of presentation. Second, our study included only overweight women, having no women falling in obesity classes 1, 2 and 3.

Our study has no statistically significant intergroup differences in post-partum complications like perineal tear and post-partum haemorrhage (PPH). The findings of other studies have been variable; some have revealed identical results, like in a study by Bjorklund et al., no intergroup difference which was statistically significant could be demonstrated [11], while others have revealed that obese women are at higher risk of PPH. According to Fyfe et al., regardless of the method of birth, overweight and obese nulliparous women had a twofold higher risk of serious PPH than women with a normal BMI. Obese women have greater rates of PPH, unrelated to the fact that they have more caesarean deliveries. In fact, a significant high-risk factor for PPH is obesity [15]. Our study could not depict increased chances of such complications possibly due to the fact that our sample size was small, with unequal distribution of patients in the two groups.

Obesity in pregnancy may adversely affect the neonatal outcome. This was reflected in the present study by a low Apgar score at 1 minute in Group B neonates as compared to neonates of Group A mothers (n=5, 14.28% vs n=4, 3.47%). Although low at 1 minute, on re-assessment at 5 minutes, the Apgar score improved and was comparable for neonates of both groups, further reflecting our finding of similar rates of NICU admissions. Similar observations were shown by Bjorklund et al., wherein no statistically significant difference was seen in the Apgar score of neonates at 5 minutes belonging to normal BMI and obese mothers [11]. However, contrasting observations were made by Khalifa et al., where newborns of obese mothers were more likely to have poor Apgar scores at 1 minute, 5 minutes and higher likelihood of NICU admissions. Their study showed the mean Apgar of neonates at 1 minute to be 8 and 7.7 in normal BMI and overweight groups, respectively, which at 5 minutes on re-assessment improved to 8.7 and 8.3 in their respective groups [6]. This was in contrast to our results, where the difference in Apgar score was insignificant at 5 minutes. One of the reasons for this may be because our study only consisted of overweight but non-obese women. Also, the Apgar score has a drawback in that it only offers information about a newborn's physiology at a particular point in time. Although it helps assess the effectiveness of resuscitation, it should not be used to assess long-term outcomes. The Apgar score calculated at 1 minute has not significantly impacted clinical outcomes over the long term. Even though it is frequently reported, the Apgar score alone should not be used as evidence of asphyxia, and its significance in outcome studies is frequently inappropriate [16].

The main limitation of the present study is a small sample size, unequal number of subjects between the groups, and lack of patients with obesity class I, II and III. Despite these limitations, the significance of our work stems from its demonstration that elevated BMI significantly affects the foeto-maternal outcome of labour. Our study's strength includes its restriction to a very specific population of overweight, singleton, full-term primigravida. Most other studies done in this area have focused on clinically obese patients, but our study enrolled only overweight females which makes it unique. 

## Conclusions

The findings of this study reveal that obesity in pregnancy significantly influences the perinatal outcomes of the mother and the foetus. Overweight primigravida women are more likely to experience less than ideal results, such as needing higher doses of PGE2 gel for induction, prolonged labour, higher frequency of caesarean section, and poor neonatal Apgar score at initial assessment. Therefore, early diagnosis of obesity in antenatal clinics and instituting interventions for weight loss can prove vital in improving foeto-maternal outcomes.
